# Development and Validation of an UHPLC-MS/MS Method for the Simultaneous Determination of 11 EU-Regulated Mycotoxins in Selected Cereals

**DOI:** 10.3390/jof8070665

**Published:** 2022-06-24

**Authors:** Marija Kovač, Ante Nevistić, Tihomir Kovač, Jurislav Babić, Antonija Šarić, Borislav Miličević, Mario Panjičko, Bojan Šarkanj

**Affiliations:** 1 Department of Food Technology, University North, Trg Dr. Žarka Dolinara 1, 48000 Koprivnica, Croatia; marija.kovac@unin.hr (M.K.); bojan.sarkanj@unin.hr (B.Š.); 2 Inspecto Ltd., Industrijska Zona Nemetin, Vukovarska Cesta 239b, 31000 Osijek, Croatia; ante.nevistic@inspecto.hr; 3 Faculty of Food Technology, Josip Juraj Strossmayer University of Osijek, Franje Kuhača 18, 31000 Osijek, Croatia; jbabic@ptfos.hr (J.B.); antonija.saric@ptfos.hr (A.Š.); borislav.milicevic@ptfos.hr (B.M.); 4 Polytechnic in Požega, Vukovarska 17, 34000 Požega, Croatia; 5 CROTEH—Sustainable Technologies Development Centre Ltd., Dragutina Golika 63, 10000 Zagreb, Croatia; mario.panjicko@croteh.eu

**Keywords:** food and feed safety, cereals, multi-mycotoxin analysis, UHPLC-MS/MS, method performance

## Abstract

The availability of reliable sensitive multi-analyte methods for unambiguous determination of mycotoxins is crucial for ensuring food and feed safety, considering their adverse health effects and (co-)occurrence in various foods. Accordingly, a multi-mycotoxin confirmatory method for simultaneous determination of 11 mycotoxins regulated in cereals within the European Union (EU) using ultra-high performance liquid chromatography coupled to tandem mass spectrometry (UHPLC-MS/MS) was developed and in-house validated to fit the EU legislation requirements for analytical methods. A simple sample preparation was based on a solid–liquid extraction using a solvent mixture acetonitrile/water/formic acid (79/20/1, *v*/*v*/*v*) and a dilution of raw extract using water/acetonitrile/formic acid (79/20/1, *v*/*v*/*v*) before instrumental analysis. Average recoveries in all three validated cereal crop types (maize, wheat, and barley), spiked at multiple levels, were found acceptable for all analytes when matrix-matched calibration was used, ranging from 63.2% to 111.2% and also showing very good repeatability, with relative standard deviations below 20%. Matrix effect (SSE) evaluation revealed maize as the most complex of the three analyzed cereal matrices, with strong SSE (<50% and >150%) recorded for all 11 analyzed mycotoxins. An additional method verification was performed through successful participation in proficiency testing schemes, with the achieved z-scores generally in the acceptable range of −2 ≤ z ≤ 2. The obtained validation results demonstrated the suitability of the developed confirmatory multi-mycotoxin UHPLC-MS/MS method based on a dilute-and-shoot principle for the simultaneous determination of low concentrations of 11 EU-regulated mycotoxins in cereals, including aflatoxins B1, B2, G1 and G2, deoxynivalenol, fumonisins B1 and B2, zearalenone, T-2 and HT-2 toxins, and ochratoxin A.

## 1. Introduction

Geographical distribution and (co-)occurrence of mycotoxins, toxic contaminants of fungal origin, in certain foodstuff and feedstuff is greatly influenced by year-to-year varying weather conditions, together with climate change effects, e.g., extreme changes in rainfall/drought episodes [1,2,3]. Given their incidence and pronounced toxicological health impact, regulations setting their threshold levels within the food and feed chain are given by European Union (EU) legislation [4,5,6,7]. The most significant mycotoxins for agricultural, primarily cereal production are aflatoxins (AFT)-B1 (AFB1), B2 (AFB2), G1 (AFG1) and G2 (AFG2), deoxynivalenol (DON), fumonisins (FUM)-B1 (FB1) and B2 (FB2), zearalenone (ZEA), T-2 and HT-2 toxins, and ochratoxin A (OTA). In order to protect the consumers’ health, i.e., ensure food and feed safety and compliance with the EU legislation, constant control of their levels is necessary. Accordingly, there is a need for highly sensitive methods/techniques to unambiguously determine these contaminants.

Considering the legislative requirements for mycotoxins in food and feed safety control and the fact that a product can be contaminated with more than one mycotoxigenic fungi that is able to produce several mycotoxins at the same time [8,9], there is a need for multi-mycotoxin methods, capable of simultaneously determining a large number of compounds. The co-occurrence of mycotoxins in agricultural products is very common [2,3,10,11], and the toxicological effects of various mycotoxins, potentially additive, synergistic or antagonistic, have not yet been sufficiently investigated. Methods employing (ultra-high performance) liquid chromatography coupled to (tandem) mass spectrometry, (UHP)LC-MS/(MS), are a good choice for the unambiguous determination of a large number of chemically distinct compounds in a relatively short analysis time [8,12,13]. Such methods are considered to be more selective and sensitive, with increased confidence in analyte identification, compared to traditional methods with conventional detectors, enabling the use of simple sample preparation procedures, saving time and reducing costs [13]. Accordingly, the use of LC–MS in the determination of low-molecular-weight contaminants at trace levels has increased considerably over the last few decades. The first validated multi-mycotoxin LC-MS/MS method was developed by Sulyok et al. (2006) [8] for the determination of 39 free and modified mycotoxins in wheat and maize, which was expanded over time to several hundred fungal and other secondary metabolites in various food and feed matrices [14,15,16,17]. Recently, in mycotoxin analytics, the focus shifted to fewer analytes in specific matrices [18]. For example, Habler et al. (2017) [19] developed a method for the determination of 12 *Fusarium* mycotoxins in beer, Šarkanj et al. (2018) [20] for the detection of the 12 most common mycotoxins in urine, Sun et al. (2019) [21] for identification and quantification of 10 emerging mycotoxins in various food matrices including sugars and beverages, and Ramö et al. (2021) [22] for the five main *Fusarium* mycotoxins in onions.

Nevertheless, in the development of such LC-MS-based multi-target methods there are several analytical challenges, including dealing with the physicochemical diversity of compounds, optimization of chromatographic separation and sample preparation procedures, and choosing sufficiently large concentration ranges to meet legislation limits for each mycotoxin, varying depending on the type of matrix of interest. To avoid loss of certain mycotoxins, a proper extraction procedure is of great importance, and thus, a minimalistic approach to sample preparation is generally sought, without the use of any purification techniques, most often the so-called dilute-and-shoot principle. However, using a simple sample preparation is likely to lead to a significant matrix effect having an impact on the method’s performance characteristics, i.e., quantification reliability.

The aim of this study was to develop and validate a fast and simple multi-mycotoxin UHPLC-MS/MS method, based on a single extraction step without clean-up, for determination of the 11 above-mentioned mycotoxins regulated by the EU legislation in cereals [4,5,6,7]. The method was validated according to the relevant EU legislation [23,24] to fulfill performance criteria and other requirements set for analytical methods.

## 2. Materials and Methods

**Chemicals and materials.** LC-MS grade acetonitrile (ACN) and methanol (MeOH) were obtained from J.T. Baker (J.T. Baker, Deventer, The Netherlands). LC-MS grade formic acid (FA) and acetic acid (HAc), LC-MS ammonium formate (AFNH_4_) and ammonium acetate (AA), were supplied by Sigma-Aldrich (Sigma-Aldrich, St. Louis, MO, USA). Ultrapure water (H_2_O) was generated by a Niro VV system (Nirosta d.o.o., Osijek, Croatia). Certified standards of mycotoxins were obtained from Romer Labs Biopure (Romer Labs, Tulln, Austria): AFT-AFB1, AFB2, AFG1 and AFG2 (2.0 μg/mL for AFB1/AFG1 0.5 μg/mL for AFB2/AFG2, lot number L17324A), DON (100 μg/mL, lot number L17194D), FUM-FB1 and FB2 (50 μg/mL, lot number L17042M), ZEA (100 μg/mL, lot number L172817Z), T-2 (100 μg/mL, lot number L171837), HT-2 (100 μg/mL, lot number L17194H), and OTA (10 μg/mL, lot number L17223A). Blank cereal samples of maize, wheat and barley were collected from Croatian fields. Proficiency testing cereal samples used for external method proving were purchased from Fapas (Fera Science Ltd., York, UK), Romer Labs (Romer Labs, Tulln, Austria) and Bipea (Bipea, Paris, France).

**Instrumental conditions.** The optimized instrumental method conditions used to determine the 11 EU-regulated mycotoxins in cereals (AFB1, AFB2, AFG1, AFG2, DON, FB1, FB2, ZEA, T-2, HT-2, and OTA) was previously described by Kovač et al. (2021) [2]. Briefly, the analysis was carried out using an UHPLC (Acquity H-Class, Waters, Milford, MA, USA) equipped with a quaternary pump system, coupled with a triple quadruple mass spectrometer (XEVO TQD, Milford, MA, USA) using an orthogonal Z-spray electrospray interface (ESI). The chromatographic separation was attained using Acquity HSS T3 reversed phase column (100 × 2.1 mm, 1.8 µm particle size) (Waters, Milford, MA, USA) maintained at 40 °C and an aqueous solution of 5 mM AFNH_4_ and MeOH, as mobile phases A and B, respectively, at a constant flow rate of 0.3 mL/min. The optimized gradient elution started with 95% A followed by a linear decrease to 50% A in 6 min and in the next 4 min to 5% A with a hold time of 5 min, afterwards switching to 95% A and column equilibration to initial conditions in the next 3 min, giving a total analysis time of 18 min. The injection volume was 10 µL. ESI-MS/MS analysis was performed in a multiple reaction monitoring (MRM) mode in positive and negative polarity, with two MS/MS transitions acquired per analyte and a dwell-time between 0.017 and 0.130 s depending on the mycotoxin. Cone voltage and collision energy values were optimized for each precursor ion and product ions, together with ionization source parameters, to achieve the highest response for all analytes (for numerical values see Section 3). The other MS and MS/MS parameters used for analysis in both positive and negative ESI, optimized in such way to provide the highest signal intensity, amounted as follows: capillary 1.5 kV (+) and 2.5 kV (−), source temperature 150 °C, desolvation temperature 350 °C, cone gas flow 50 L/h and desolvation gas flow 650 L/h (both gases nitrogen). For collision, argon was used with a pressure of approximately 4.0 × 10^−3^ mbar in the collision cell.

The optimization of MS and MS/MS parameters was performed by a combined infusion of an analytical standard of each mycotoxin (1 μg/mL solution prepared in ACN) at a flow rate of 20 μL/min and mobile phases (aqueous 5 mM AFNH_4_/MeOH, 50/50, *v*/*v*) at a flow rate of 0.1 mL/min into a mass spectrometer, working in both positive and negative ESI. MS and MS/MS spectra were recorded under different conditions to obtain at least one precursor ion at the optimal cone voltage, and individual ion fragments (product ions) for each analyte at optimal collision energies. The most abundant product ion was chosen for quantification, and the other for confirmation, afterwards all were compared with literature data to authenticate.

**Figure 1:**
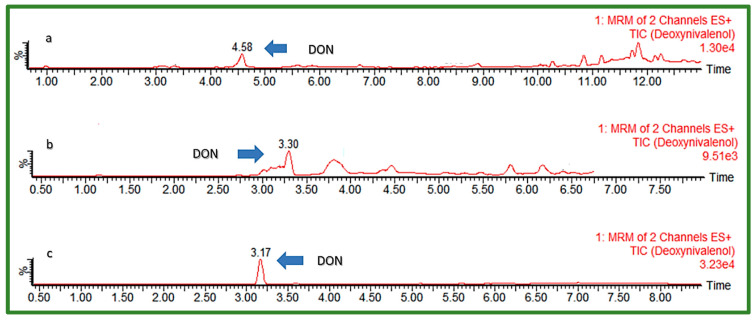
UHPLC-MS/MS chromatogram of early eluting DON (50 ng/mL) using different C18 columns: (**a**) Phenomenex Kinetex EVO C18, (**b**) Restek Aqueous C18, (**c**) Waters Acquity HSS T3.

For the optimization of chromatographic separation, the combination of H_2_O and organic solvents MeOH and ACN in different ratios varying between 0 and 25% of the organic phase content in the initial gradient composition was investigated (Table 1). The addition of modifiers to mobile phases, such as AFNH_4_, AA, FA, and Hac, was also examined. Furthermore, different modifications of the C18 analytical columns from different manufacturers were used for chromatographic separation testing: Kinetex EVO C18 (150 × 2.1 mm, I.D. 1.7 μm) (Phenomenex, Torrance, CA, USA), Ultra Aqueous C18 (100 × 2.1 mm, I.D. 3 μm) (Restek, Bellefonte, PA, USA), and Acquity HSS T3 (100 × 2.1 mm a I.D. 1.8 μm) (Waters, Milford, MA, USA). Elution gradient 4, shown in Table 1, with 5 mM aqueous solution of AFNH_4_ (A) and MeOH (B) as mobile phases, were used to test all three columns, to choose the best one for further optimization. Matrices of extracted cereal samples spiked with all EU-regulated mycotoxins were used for the investigation by injection into a UHPLC-MS/MS system, after which peak shape and obtained response were observed for each analyte. The column with the best performance was further tested to find the most suitable flow rate, injection volume, and column temperature, as well as final mobile phase gradient. UHPLC-MS/MS chromatograms of a “problematic” early eluting polar compound DON using different C18 columns are presented in Figure 1.

**Calibration and daily quality control.** For quantification purposes external (matrix-matched) calibration was used for each cereal type. Therefore, a multi-analyte (combined) standard solution was prepared by mixing appropriate volumes of each certified mycotoxin standard stated above (25 µL AFT, 50 µL DON, 75 µL FUM, 7.5 µL ZEA, 2.5 µL T-2, HT-2, and OTA) and ACN (835 µL). Afterwards, combined standard solution was diluted 1:1 with ACN/H_2_O (50/50, *v*/*v*) or suitable blank cereal matrix to provide working solutions (calibrants) in different concentrations (400×, 200×, 100×, 50×, 25×, and 10× diluted) to match the expected analyte concentration in the diluted (final) extracts.

Prior to the analysis, a blank solvent mixture, was injected to ensure system`s equilibration, followed by the set of calibrants and another two blank injections before samples for preventing any carry-over from the most concentrated calibrant. Internal method quality control was conducted by performing the recovery experiments by spiking cereals within the sample batch, correcting the measured mycotoxin concentrations for recovery when outside the allowed range (90–110%) for mycotoxins by Commission Regulation (EC) No. 401/2006 [23]. In addition, at the end of each batch a calibrant was injected as a control of calibration curve suitability, ensuring that not too much time had passed from the calibrant injection.

Furthermore, for analyte identification, according to the criteria of Commission Decision (EC) No. 657/2002 [24], the relative retention time of the certain mycotoxin in the sample solution had to correspond to the one in the standard solution at a tolerance of ±2.5%, while the ratio of relative ion intensities of the analyte in the sample had to match the ratios in standard solution from the same measurement sequence with a permitted tolerance of ±30%.

**Extraction procedure and spiking.** A sample portion of 5 g was weighed into polypropylene centrifuge tubes and extracted by solvent mixture ACN/H_2_O/FA (79/20/1, *v*/*v*/*v*) using mechanical shaker for 90 min and subsequently centrifuged for 5 min at 3000× *g* at room temperature using a Restek Q-sep 3000 centrifuge (Restek, Bellefonte, PA, USA). Aliquot of the raw extract was diluted using the same volume of ACN/H_2_O/FA (20/79/1, *v*/*v*/*v*) and appropriately mixed. Diluted extract was afterwards filtered through a 0.22 µm nylon filter and without further clean-up injected into an UHPLC-MS/MS system. To economize the usage of the analytical standards, the spiking protocol was miniaturized, and 1 g cereal samples were used for the validation experiments. Ground cereals were spiked by adding the appropriate amount of combined standard solution, left overnight at a room temperature to equilibrate, and subsequently extracted using optimized extraction solvent.

To perform optimization of the extraction solvent, different mixtures of H_2_O and organic solvents with and without the addition of FA or HAc were tested. Therefore, solvent extraction mixtures including ACN/H_2_O (80/20, *v*/*v*) and ACN/H_2_O/HAc (79/20/1, *v*/*v*/*v*), proposed by Frenich et al. (2009) [25] and Malachová et al. (2014) [16], respectively, were tested, as well as their modifications. Moreover, the influence of different extraction time on the extraction efficiency of individual mycotoxins was tested, to select the most favorable one for the extraction of all analytes.

**Method validation and performance characteristics.** In addition to the internal need for validation of the method, i.e., the professional responsibility of the analyst to give credible measurement results, there are legislation requirements for demonstrating a method’s compliance with the criteria applicable for the relevant performance characteristics. General guidelines on the performance of analytical methods and interpretation of results are given in Commission Decision 2002/657/EC [24]. For mycotoxins specifically, Commission Regulation (EC) No. 401/2006 [23] sets the methods of sampling and analysis for the official control of the levels of mycotoxins in foodstuffs, and among them, method performance criteria (Table 2) for each mycotoxin regulated by EU legislation in certain food- and feedstuffs [4,5,6,7]. In addition, Commission Decision 2002/657/EC [24] also establishes the term “confirmatory method”, referring to methods that provide full or complementary information enabling the substance to be unequivocally identified and if necessary quantified at the level of interest.

Within the in-house validation, method performance characteristics including selectivity, linearity (calibration curves), sensitivity (limits of detection and quantification), trueness (method recovery), and precision (repeatability and reproducibility—via proficiency testing) were evaluated. For method sensitivity evaluation, blank samples of each crop type were fortified at the level of targeted limit of detection (LOD) and quantification (LOQ), with the signal to noise ratio (S/N) calculation conducted with respect to the confirmatory (LOD) and quantitative (LOQ) MRM transition. The values were regarded as acceptable if the ratios were over 3 and over 10, respectively. Linearity was tested for neat solvent calibrants and matrix-matched calibrants by instrumental measuring in triplicate to create calibration curves and to evaluate the coefficient of determination (R^2^), which was to meet a criterion of at least 0.99. For other method performance characteristics, fortification of blank cereal samples at three different spiking levels was chosen to cover the respective limits of quantification of each compound, legislation (threshold) limits of regulated mycotoxins in certain cereal crop types, and the level of one-and-a-half times the legislation limit, as stated at the Table 3. Each fortification level was spiked in six replicates, while each replicate was measured three times. Results were determined using neat solvent calibration and matrix-matched calibration prepared for each cereal type separately.

Additionally, although not covered by the relevant EU legislation for mycotoxins, the matrix effect was also assessed. The matrix effect may have a significantly negative impact on LC-MS analysis, i.e., the ionization efficiency of the analyte, particularly when ESI is used, causing suppression or enhancement of the analyte signal, thus affecting quantification, possibly leading to incorrect results, especially when neat solvent standards are used. The extent of the matrix effect depends on the type of the matrix itself and variability between samples of the same type, preparation procedure and chromatographic and MS analysis conditions, as well as chemical properties of the analyte [13,17]. There are several ways to reduce or eliminate the influence of the matrix, including modification of MS analysis conditions, optimization of sample preparation, modification of chromatographic conditions, and use of alternative calibration procedures, among them the most common external matrix-matched calibration curves, the standard addition approach, or the increasingly more popular stable isotope dilution technique [8,13,26,27]. Therefore, the matrix effect, i.e., matrix-induced enhancement or suppression, but also associated characteristics such as extraction efficiency and apparent (absolute) recovery were evaluated, to separately evaluate sample preparation step and measurement process [17,21]. For compounds for which a significant matrix effect was established during the validation experiments, the use of matrix-matched calibration for mycotoxin quantification was investigated.

**Data evaluation.** For peak integration purposes, linear, 1/x weighted calibration curves were constructed from data obtained by measuring in triplicate each sample type (spiked samples, neat solvent calibrants, and matrix-matched calibrants) using MassLynx and TargetLynx software (v. 4.1., Waters, Milford, MA, USA) to evaluate the linearity of the method. The same software was used to determine S/N for each mycotoxin in order to evaluate LOD and LOQ. Data evaluation for other method performance characteristics was performed using Microsoft Excel 2016 (Microsoft, Redmond, WA, USA) and spiking experiments for each cereal type (maize, wheat, and barley) described above. Method recovery (R) was calculated for each spiking level and replicate according to Equation (1), after which the average method recovery for each mycotoxin was expressed.

(1) $$R\%=\frac{\mathrm{measured}\;\mathrm{concentration}\;\mathrm{of}\;\mathrm{spiked}\;\mathrm{sample}}{\mathrm{fortification}\;\mathrm{level}}\times 100$$

The method repeatability was expressed as the relative standard deviation (RSD) in percentage, calculated from the measurements of six spiked replicates for each cereal crop and fortification level (repeatability of sample preparation, RSD_P_ and repeatability of measurements, RSD_M_).

Method reproducibility was proved through participation in proficiency testing (PT) schemes. PT samples of various cereal matrices were obtained from PT providers including Fapas, Romer Labs, and Bipea. The evaluation of participation is given by the so-called z-score, calculated by the provider as follows:

(2) $$z=\frac{\mathrm{reported}\;\mathrm{value}-\mathrm{PT}\;\mathrm{assigned}\;\mathrm{value}}{\mathrm{PT}\;\mathrm{standard}\;\mathrm{deviation}}\times 100$$

Matrix-induced enhancement or suppression (SSE) was assessed by comparing the average area of analyte in matrix-matched standard and in neat solvent standard, both at targeted LOQ value, using Equation (3):

(3) $${\mathrm{SSE}}_{A}\%=\frac{\mathrm{average}\;\mathrm{area}\;\left(\mathrm{matrix}-\mathrm{matched}\;\mathrm{standard}\right)}{\mathrm{average}\;\mathrm{area}\;\left(\mathrm{neat}\;\mathrm{solvent}\;\mathrm{standard}\right)}\times 100$$

For better understanding of the matrix effect, SSE was also estimated by comparing the slopes of matrix-matched calibration curves to solvent curves, according to Equation (4):

(4) $${\mathrm{SSE}}_{S}\%=\frac{\mathrm{slope}\;\left(\mathrm{matrix}-\mathrm{matched}\;\mathrm{curve}\right)}{\mathrm{slope}\;\left(\mathrm{neat}\;\mathrm{solvent}\;\mathrm{curve}\right)}\times 100$$

Extraction efficiency (R_E_) and apparent recovery (R_A_) were calculated by comparing the average area of analyte in a spiked sample and in a matrix-matched standard or neat solvent standard, respectively:

(5) $$R_{E}\%=\frac{\mathrm{average}\;\mathrm{area}\;\left(\mathrm{spiked}\;\mathrm{sample}\right)}{\mathrm{average}\;\mathrm{area}\;\left(\mathrm{matrix}-\mathrm{matched}\;\mathrm{standard}\right)}\times 100$$

(6) $$R_{A}\%=\frac{\mathrm{average}\;\mathrm{area}\;\left(\mathrm{spiked}\;\mathrm{sample}\right)}{\mathrm{average}\;\mathrm{area}\;\left(\mathrm{neat}\;\mathrm{solvent}\;\mathrm{standard}\right)}\times 100$$

## 3. Results and Discussion

### 3.1. Method Development—UHPLC-MS/MS Optimization

In order to select and optimize the MS and MS/MS detection parameters for each analyte, MS spectra of each compound were acquired to obtain at least one precursor ion and the optimal cone voltage, while MS/MS spectra (product ion scan) were recorded to obtain quantification and confirmation product ions, characteristic for a particular mycotoxin. By selecting one precursor ion and two product ions per analyte, 4.0 identification points were achieved, thus satisfying a minimum of 3.0 identification points for the interpretation of data required by Commission Decision (EC) No. 657/2002 [24] in the case of mycotoxins. The optimized MRM parameters (precursor and products ions, cone voltage, and collision energies) selected for the detection are shown in Table 4. For most of the analytes, a protonated adduct [M+H]^+^ was selected as a precursor ion, while for T-2 and HT-2 toxins a more abundant ammonium adduct [M+NH_4_]^+^ was selected. Positive ESI was chosen for all analytes, except for ZEA, for which negative ESI and deprotonated adduct [M–H]^−^ were selected, due to the appearance of interference in some matrices when using positive ESI. The dwell-time used for the analysis was between 0.017 and 0.130 s, depending on the analyte, which provided enough points per peak to enable necessary sensitivity and reproducibility of the quantitative results. The selected MRM transitions for the analyzed regulated mycotoxins coincide with the transitions in the works of other authors [8,25,28,29,30]. On the other hand, given the instruments are of various manufacturers, the optimized MS conditions such as cone voltage and collision energy differ significantly, as expected.

Given the chemical diversity of mycotoxins, i.e., opposed polarity or acidity [8], it was necessary to find the most suitable chromatographic separation conditions, including mobile phase composition, elution gradient and UHPLC column type, in order to achieve the optimal peak shapes for all compounds, required sensitivity, and other method performance characteristics, with an acceptable duration of the analysis. The use of MeOH instead of ACN as the mobile phase enabled higher analyte sensitivity (response) in general, especially for trichothecenes.

The addition of modifiers, in this case AFNH_4_ as it gave higher responses than AA, was necessary to suppress the formation of undesirable sodium adducts, i.e., to enable the formation of desirable [M+NH_4_]^+^ adducts that allowed higher intensities and required LOQ values in all types of matrices, especially for T-2 and HT-2 toxins, important for possible method application in the analysis of cereal products intended for direct consumption, such as bread, pasta, and snack products, with lower indicative levels [5].

**Figure 2:**
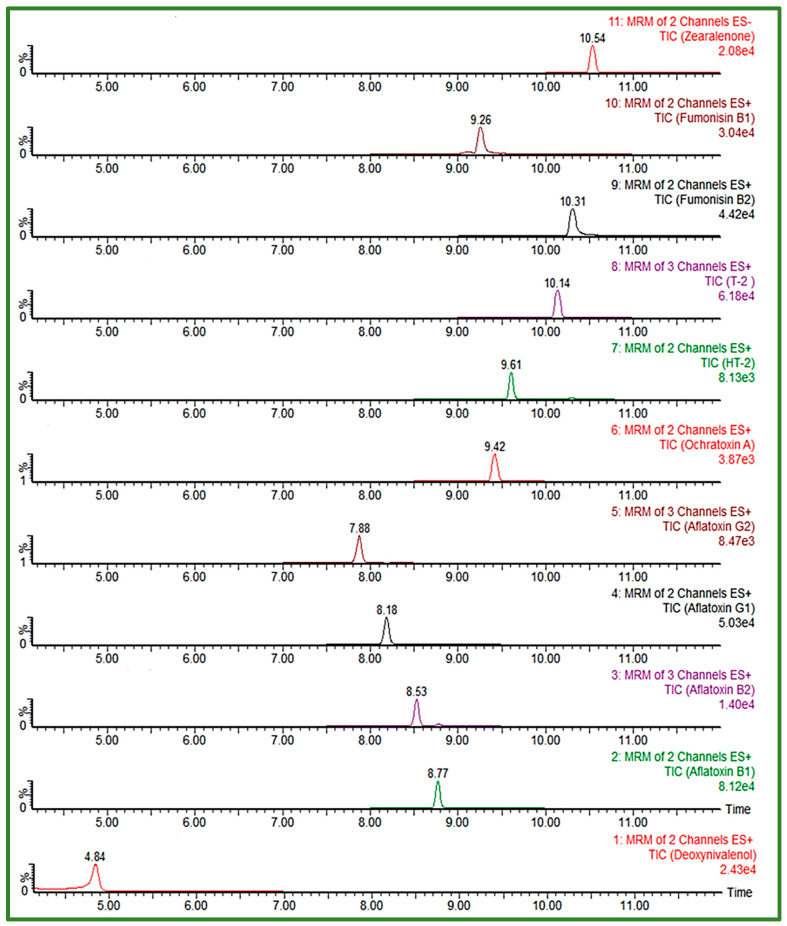
UHPLC-MS/MS chromatogram of 11 EU-regulated mycotoxins in spiked solvent (AFB1/AFG1 0.5 ng/mL, AFB2/AFG2 0.125 ng/mL, DON 50 ng/mL, FB1/FB2 37.5 ng/mL, ZEA 7.5 ng/mL, T-2/HT-2 2.5 ng/mL and OTA 0.25 ng/mL) obtained using Acquity HSS T3 column under optimized conditions.

In order to achieve satisfactory separation and peak shape for FUM, slightly acid chromatographic conditions had to be ensured, given the four carboxyl groups in the FUM molecule structure [8,25], thus gradients with the addition of 0.1% FA or HAc to the mobile phases were also tested. An important segment of the separation was the proportion of the organic phase in the initial conditions of the gradient, having the effect on peak shape of the early eluting compounds. The Acquity HSS T3 column (100 × 2.1 mm, I.D. 1.8 μm) proved to be very useful in the separation of polar, early eluting compounds, as it gave the best peak shape for all analytes despite their chemical diversity, using 5 mM aqueous AFNH_4_ (pH 6.4) and MeOH, with a gradient start at 95% of aqueous mobile phase, at a constant flow rate of 0.3 mL/min during the 18 min run time. Considering the selected composition of the mobile phase, elution gradient and flow rate, the column temperature of 40 °C was chosen to enable UHPLC system’s pressure to be within the allowed values (up to 15,000 psi). From the tested injection volumes, 10 μL was selected as the final one, with an acceptable response and peak shape for all analytes. The gradient, optimized to increase the separation efficiency of analytes eluting close to the polarity change of the used mobile phase is presented in Table 5. The obtained UHPLC-MS/MS chromatogram of 11 EU-regulated mycotoxins in cereals obtained using an Acquity HSS T3 column and optimized chromatographic conditions is shown in Figure 2.

Looking at the work of other authors, mainly UHPLC, but also HPLC analytical columns with variations of C18 packing materials were used for chromatographic separation of analytes, maintained at 25–40 °C, with a mobile phase flow of 0.3–1.0 mL/min, mostly consisting of H_2_O and MeOH with the addition of modifiers (AFNH_4_, AA, FA, HAc, etc.) [8,16,25,28,29,30,31,32,33], confirming MeOH as more suitable for mycotoxin chromatographic separation. In addition, a relatively high percentage (≥75%) of the aqueous mobile phase in the initial conditions was generally maintained, and the injection volume was kept at no more than 20 µL, depending on the column type.

### 3.2. Method Development—Sample Preparation

As the chemical diversity of the analytes affects every step of the multi-mycotoxin method development, including the extraction step, the composition of the extraction solvent mixture is often critical for preserving all the compounds of interest, most frequently mentioned for FUM [8,13,29,34]. In addition to the extraction conditions, the clean-up procedure is also to be considered, and is often omitted in order to save analytes, as well as time and money, and to reduce waste [13]. Thus, prior to the UHPLC-MS/MS analysis, a simple sample preparation procedure was performed, including liquid–solid extraction, followed by dilution of the raw extract. For the preparation of the sample, different extraction conditions were tested, with regards to the composition of the extraction solvent (different ratios of ACN and H_2_O, addition of FA or HAc). A mixture of water with high levels of organic solvent (MeOH or ACN, over 70%) is suitable for the extraction of most mycotoxins, but in the case of hydrophilic FUM, a higher water content in the extraction solvent and/or a lower pH of the solvent is desirable [8,9,29,34,35]. Therefore, the addition of 1% acid, namely FA, was selected for extraction, as there was no significant difference compared to the addition of HAc. The lower proportion of the aqueous phase in the chosen extraction solvent, ACN/H_2_O/FA (79/20/1, *v*/*v*/*v*), was also compensated for by an extraction time of 90 min at which optimal recovery was achieved for all analytes. The obtained raw extract was diluted with the same volume of a solvent mixture, ACN/H_2_O/FA (20/79/1, *v*/*v*/*v*), in order to eliminate the possible matrix effect on individual compounds [8]. In this way, the content of the organic phase that affects the deformed appearance of the peaks of compounds eluting early from the analytical column, such as DON, was also reduced.

The dilute-and-shoot principle of sample preparation in mycotoxin determination has also been applied by other authors [8,16,25,30,31], performed using solvent mixtures consisted of H_2_0, ACN and/or MeOH, and FA or HAc, but often in combination with sample extract evaporation and reconstitution. The Quick, Easy, Cheap, Effective, Rugged, and Safe (QuEChERS) approach was also frequently employed [28,32,33]), consisting of acidic acetonitrile extraction/salt mixture partitioning and clean-up by dispersive solid phase extraction, and in certain cases followed by evaporation and reconstitution using a suitable solvent. Compared to the sample preparation methods developed by the aforementioned authors for the analysis of a similar scope of analytes and matrices [28,29,30,33], the method developed in this work has the same or shorter extraction time for the same analytes, a cheaper preparation procedure and shorter overall sample preparation time, since it does not involve evaporation or reconstitution steps.

### 3.3. Method Performance

Method validation, i.e., proving the method’s performance, is a key activity in analytics in general, and is necessary for gathering reliable and comparable measurement results. Validation experiments are generally planned individually, depending on the analyte, method, and matrix, and their scope must be sufficient to meet the application scope of the method. Validation procedure is therefore a compromise between cost, risks, and technical possibilities: the more important and complex the methods are, the more extensive the validation process is. Accordingly, within this work the following method performance characteristics were evaluated for each cereal type: selectivity, sensitivity, trueness, and precision, along with the matrix effect.

Method selectivity was studied at the beginning of the UHPLC-MS/MS method development process, by the analysis of blank cereal samples, spiked cereal samples, and blank solvent. The absence of chromatographic signal at the correct retention time and MRM transitions when blanks were applied, indicated proper selectivity of the developed multi-mycotoxin method. For that reason, for all analytes positive ESI was selected, except for ZEA for which negative ESI was chosen.

LOD and LOQ, evaluated by using the spiked cereal samples, were used to establish the method sensitivity. The achieved LOD and LOQ values stated in Table 6, together with the achieved determination coefficients of calibration curves conforming to the criteria ≥0.99 and established concentration ranges for all analytes were evaluated as acceptable, since satisfying the requirements of sufficient S/N ratio and occurrence of confirmation ions. The lowest LOD and LOQ were achieved for AFB1 and AFG2 (both 0.5 µg/kg), and the highest for DON (200 µg/kg). Even though somewhat higher than the values reported by other authors [28,29,30], when compared to the EU legislation threshold limits, the obtained LOQ values were lower for all analytes, indicating the suitability of the developed method for determining low concentrations of the selected EU-regulated mycotoxins in cereals, and also products thereof.

Method trueness and precision were proven within the laboratory through recovery experiments as repeatability of six spiked replicates at the three fortification levels, with each replicate being measured instrumentally three times. Calculated average method recovery, repeatability of sample preparation and repeatability of measurements for all analytes and cereal matrices obtained using both calibration approaches are presented in Table 7, Table 8 and Table 9. For the most of the analytes and analyzed cereals the achieved method recovery values were within the legislation requirements [23] using at least one calibration approach. The highest and lowest accepted individual method recovery values were observed in maize for HT-2 and DON, amounting to 122.6% at spike level 3 and 66.4% at spike level 1, respectively. For FB1 and FB2 average recoveries in all three cereal matrices, especially maize, were significantly higher than allowed when neat solvent calibration was applied; however, when using matrix-matched calibration, recoveries were within the allowed range at all three spike levels. Average method recovery for OTA in barley, even though slightly outside the allowed range (<70%), was still considered acceptable, since satisfying method recovery was achieved at the method threshold limit of LOQ. Furthermore, the obtained validation results also showed very good repeatability of instrument measurement and sample preparation at all spiking levels, with the achieved values of RSD_M_ and RSD_P_ for all analytes and cereal matrices being generally significantly lower than the set criterion of 20%, which is the lowest RSD value permitted by EU legislation [23] for a mycotoxin in cereals. Since matrix effect depends on the combination of analyte and matrix co-extractives, as already stated before, various SSE values for various mycotoxins were obtained in each cereal crop type. As relevant legislation for mycotoxins does not provide an acceptable SSE range, values suggested by Sulyok et al. (2020) [17] were used for SSE evaluation: SSE was regarded as soft in the range 80–100% (soft suppression) and 100–120% (soft enhancement), as moderate in the range 50–80% (medium suppression) and 120–150% (medium enhancement), and as strong SSE at values <50% (strong suppression) and >150% (strong enhancement). Maize proved to be the most complex of the three analyzed cereal matrices, with strong SSE being recorded for all 11 analyzed mycotoxins, while for the other two matrices moderate SSE was the most common. For better understanding of the matrix effect, SSE was estimated using both area and slope data (Equations (3) and (4)) and for certain mycotoxins significant differences were observed in SSE_A_ and SSE_S_ values. For example, SSE_S_ did not indicate a significant matrix effect for HT-2 in wheat, amounting to 98.8%, while SSE_A_ revealed signal enhancement, amounting to 210.9%.

In the case where these two values differ, SSE_A_ is taken into account, since it is estimated at the method threshold value of LOQ where the interferences are considered to be the strongest, thus giving a more accurate insight on the real extent of the matrix effect. The use of SSE_A_ equation instead of SSE_S_ was also suggested by Malachová et al. (2014) [16] and used by other authors, e.g., Sulyok et al. (2020) [17] and Sun et al. (2019) [21].

**Figure 3:**
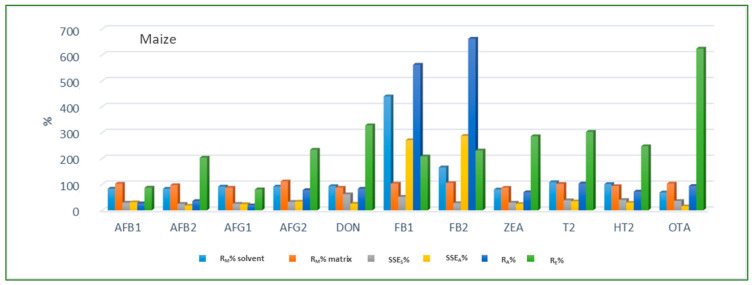
Method recovery (R_M_), matrix effect (SSE_S_-slope, SSE_A_-area), apparent recovery (R_A_) and extraction efficiency (R_E_) in maize.

**Figure 4:**
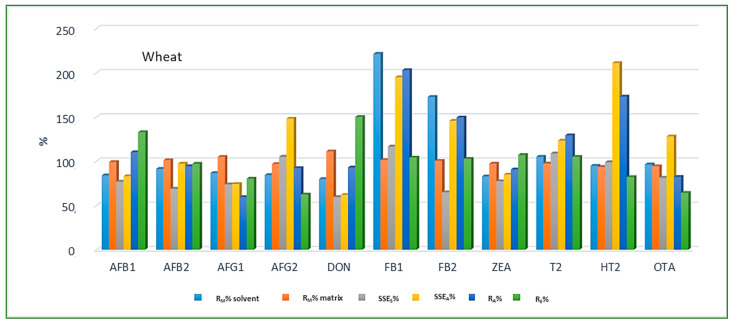
Method recovery (R_M_), matrix effect (SSE_S_-slope, SSE_A_-area), apparent recovery (R_A_) and extraction efficiency (R_E_) in wheat.

**Figure 5:**
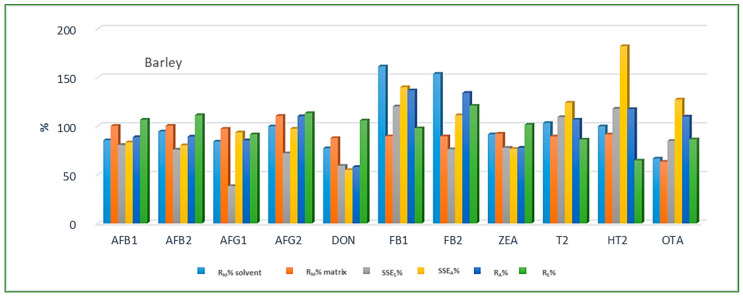
Method recovery (R_M_), matrix effect (SSE_S_-slope, SSE_A_-area), apparent recovery (R_A_) and extraction efficiency (R_E_) in barley.

Looking at the average SSE values determined for each cereal crop type, maize showed the highest analyte signal suppression with an average SSE_A_ value of 71.9% (15.0–287.2%), while in wheat and barley signal enhancement was observed with the average SSE_A_ amounting to 122.9% (62.0–210.9%) and 106.3% (54.8–181.9%), respectively. When considering the average SSE_A_ values for each mycotoxin in all three cereal matrices, the highest suppression was observed for DON (47.4%), while the highest enhancement was recorded for FB1 (201.8%). All calculated percentages of the matrix effect, extraction efficiency, and apparent recovery for each mycotoxin and cereal crop type are shown in Figure 3, Figure 4 and Figure 5, demonstrating the need for matrix effect compensation, in our case the use of matrix-matched calibration. Nevertheless, consistently high signal enhancement (strong or moderate) in all three crop types has been observed for FB1 and FB2, which is unlikely the result of the matrix effect in the narrow sense, i.e., the ESI process in the ion source, but probably the consequence of other factors, such as the matrix acting as a protectant against analyte degradation during the analytical procedure leading to losses of the analyte in the absence of matrix, as already reported for other compounds [13,17]. Furthermore, unusually high extraction recoveries were achieved for mycotoxins in maize (80.8–624.4%), compared to the other two validated matrices, wheat (62.3–150.1%) and barley (64.5–113.2%), which may be the result of the applied extraction conditions, i.e., formic acid in the extraction solvent instead of the weaker, commonly used acetic acid, possibly causing the cleavage of the bonds between maize macromolecules (starch) and mycotoxins, thus releasing entrapped mycotoxins from their hidden forms [36,37,38,39], consequently contributing to the (result) overestimation.

Method reproducibility was evaluated by participating in interlaboratory comparison tests, i.e., proficiency tests, using matrices of different cereals and products thereof. Generally, z-scores within the range −2 ≤ z ≤ 2 are considered as satisfactory, results 2 < |z| ≤ 3 are considered to be a “gray” zone or a warning signal, while results |z| > 3 are considered as unsatisfactory, requiring measures to find the cause of the problem. In our case, all achieved z-scores for analyzed analytes in individual food and feed samples, presented in Table 10, met the set criterion −2 ≤ z ≤ 2, except for FB1 in cereal-based baby food (maize-based) Bipea 12-3931 for which a “gray” zone z-score of 2.41 was achieved, probably as a consequence of the issue(s) addressed above.

In addition, this developed and validated UHPLC-MS/MS multi-mycotoxin method based on the dilute-and-shoot principle was successfully employed for EU-regulated mycotoxin determination in unprocessed cereal crops grown in Croatian fields, as already published by Kovač et al. (2021; 2022) [2,3]. A total of 119 cereal samples from the 2016 harvest were analyzed during the cereal multi-contaminant investigation, revealing 8.5% of samples to be non-compliant with the relevant EU mycotoxin legislation, with *Fusarium* mycotoxin DON being the most frequently occurring (73.7%) [2]. In the study by Kovač et al. (2022) [3] the method was used to analyze 209 cereal samples (maize, wheat, barley, oats, and rye) from 2016 and 2017 harvests from all Croatian counties, confirming the *Fusarium* mycotoxins as the main contaminants of Croatian cereals and revealing generally high mycotoxin co-occurrence in Croatian cereals (50.0% in 2016 and 33.7% in 2017), but also a correlation between year-to-year weather conditions and mycotoxin incidence, thus emphasizing the need for continuous mycotoxin control in a climate change-affected world.

## 4. Conclusions

A fast and simple confirmatory multi-mycotoxin UHPLC-MS/MS method, based on a liquid–solid extraction, followed by the dilution of the raw extract, was developed for determination of 11 mycotoxins (AFB1, AFB2, AFG1, AFG2, DON, ZEA, FB1, FB2, T-2, HT-2, and OTA) regulated by the EU legislation in cereals, and successfully in-house validated using three cereal matrices, including maize, wheat, and barley, to prove its compliance with EU requirements set for the analytical methods.

Validation experiments were conducted within the laboratory, under optimized conditions for sample preparation and UHPLC-(ESI)-MS/MS determination, by spiking blank cereal samples on multiple levels, followed by the method of external verification through participation in PT schemes. Although not encompassed within the legislation requirements but known to have an impact on method reliability, the matrix effect was also evaluated, revealing maize as the trickiest matrix, having the strongest effect of suppressing/enhancing the analyte signal. Even though a dilution step was used, matrix-matched calibration was necessary for correcting the remaining matrix effect.

In conclusion, the obtained validation results demonstrated suitability of the developed multi-mycotoxin UHPLC-MS/MS method for the simultaneous determination of low concentrations of the selected EU-regulated mycotoxins in cereals, thus confirming the method as a useful tool for ensuring food and feed safety, contributing to public health.

## Figures and Tables

**Table 1:** Tested elution gradients and mobile phase composition.

^1^	**Time min**	**A%**	**B%**	^2^	**Time min**	**A%**	**B%**	^3^	**Time min**	**A%**	**B%**	^4^	**Time min**	**A%**	**B%**
	0.0	98	2		0.0	95	5		0.0	95	5		0.0	75 *	25
	1.5	98	2		6.0	50	50		6.0	50	50		3.0	25	75
	6.0	30	70		10.0	5	95		10.0	5	95		5.0	0	100
	6.1	10	90		11.5	5	95		15.0	5	95		6.5	0	100
	7.0	10	90		11.6	95	5		15.1	95	5		7.5	75	25
	7.1	98	2		14.0	95	5		18.0	95	5		8.5	75	25
	9.0	98	2												
		0.5 mL/min				0.3 mL/min				0.3 mL/min				0.35 mL/min	

^1^ 0.1% FA in H_2_O (A) and 0.1% FA in can (B). ^2^ 2 mM AA + 0.1% FA in H_2_O (A) and 2 mM AA + 0.1% FA in MeOH (B). ^3^ 4 mM AFNH_4_ + 0.1% FA in H_2_O (A) and 4 mM AFNH_4_ + 0.1% FA in MeOH (B); 5 mM AFNH_4_ in H_2_O (A) and MeOH (B). ^4^ 5 mM AFNH_4_ in H_2_O (A) and MeOH (B). * Start gradient of 100% A also tested.

**Table 2:** Selected performance criteria values (trueness, precision) according to Commission Regulation (EC) No. 401/2006 [23].

Compound	Concentration Level µg/kg	Method Recovery (R) %	Precision Repeatability (RSD_r_) %
AFT (AFB1, AFB2, AFG1, AFG2)	<1	50–120	0.66 times Reproducibility (RSD_R_) derived from Horwitz equation at the concentration of interest
	1–10	70–110	
	>10	80–110	
DON	>100	60–110	≤20
	≥500	70–120	≤20
FUM (FB1, FB2)	≤500	60–120	≤30
	>500	70–110	≤20
ZEA	≤50	60–120	≤40
	>50	70–110	≤25
T-2/HT-2	15–250	60–130	≤30
	>250	60–130	≤25
OTA	<1	50–120	≤40
	≥1	70–110	≤20

**Table 3:** Fortification levels for each mycotoxin and cereal type.

	Maize µg/kg			Wheat µg/kg			Barley µg/kg		
Analyte	Level 1	Level 2	Level 3	Level 1	Level 2	Level 3	Level 1	Level 2	Level 3
AFB1	1.0	5.0	7.5	1.0	2.0	3.0	1.0	2.0	3.0
AFB2	0.5	1.25	1.875	0.5	0.75	1.5	0.5	0.75	1.5
AFG1	1.0	5.0	7.5	1.0	2.0	3.0	1.0	2.0	3.0
AFG2	0.5	1.25	1.875	0.5	0.75	1.5	0.5	0.75	1.5
DON	200	1750	2625	200	1250	1875	200	1250	1875
FB1	150	2000	3000	150	1000	1500	150	1000	1500
FB2	150	2000	3000	150	1000	1500	150	1000	1500
ZEA	30	350	525	30	100	150	30	100	150
T-2	10	100	150	10	100	150	10	100	150
HT-2	10	100	150	10	100	150	10	100	150
OTA	1.0	5.0	7.5	1.0	5.0	7.5	1.0	5.0	7.5

**Table 4:** MS/MS parameters under optimized conditions.

Mycotoxin	Precursor Ion m/z	Cone Voltage V	Collision Energy V	Product Ions * m/z
AFB1	313.0 [M+H]^+^	60	38	**241.0**
			23	285.0
AFB2	315.0 [M+H]^+^	60	30	**259.0**
			25	287.0
AFG1	329.0 [M+H]^+^	60	28	**243.0**
			24	311.0
AFG2	331.0 [M+H]^+^	60	24	**313.0**
			28	245.0
DON	297.0 [M+H]^+^	25	12	**231.0**
			10	249.0
FB1	722.4 [M+H]^+^	50	40	**334.3**
			40	352.3
FB2	706.4 [M+H]^+^	50	40	**336.2**
			40	318.2
ZEA	317.1 [M–H]^−^	−58	30	**131.0**
			20	175.0
T-2	484.7 [M+NH_4_]^+^	25	20	**185.0**
			25	215.0
HT-2	442.6 [M+NH_4_]^+^	25	10	**263.4**
			15	215.3
OTA	404.1 [M+H]^+^	30	24	**239.0**
			14	358.0

* Quantification ions in bold.

**Table 5:** The optimized elution gradient for chromatographic separation.

Time min	Mobile Phase A% 5 mM AFNH_4_ in H_2_O	Mobile Phase B% MeOH
0.0	95	5
6.0	50	50
10.0	5	95
15.0	5	95
15.1	95	5
18.0	95	5

**Table 6:** Method performance characteristics in neat solvent and cereal matrices.

Analyte	Curve Type	Equation	Concentration Range [µg/kg]	Linearity R^2^	LOD µg/kg	LOQ µg/kg
AFB1	Solvent	y = 4233.3x − 151.78	1.0–16	0.9966	0.30	1.0
	Wheat	y = 3255.2x − 33.549		0.9985		
	Maize	y = 1243.3x − 19.301		0.9993		
	Barley	y = 3405.3x − 97.552		0.9995		
AFB2	Solvent	y = 3330.9x − 92.654	0.5–10	0.9995	0.15	0.5
	Wheat	y = 2294.8x − 0.6601		0.9998		
	Maize	y = 821.52x − 31.61		0.9995		
	Barley	y = 2520.2x − 32.659		0.9998		
AFG1	Solvent	y = 3933.1x − 31.126	1.0–16	0.9976	0.30	1.0
	Wheat	y = 2902x + 27.304		0.9981		
	Maize	y = 976.96x − 14.994		1.0000		
	Barley	y = 1502.5x − 145.4		0.9989		
AFG2	Solvent	y = 3330.9x − 92.654	0.5–10	0.9995	0.15	0.5
	Wheat	y = 3502.6x − 9.2733		0.9997		
	Maize	y = 1075.8 − 21.436		0.9999		
	Barley	y = 2394.8x − 0.6601		0.9998		
DON	Solvent	y = 14.19x + 40.633	200–4000	0.9999	61	200
	Wheat	y = 8.4400x + 3.6843		0.9992		
	Maize	y = 8.7478x + 11.739		0.9997		
	Barley	y = 8.3797x − 2.8576		0.9999		
FB1	Solvent	y = 198.06x − 3113	150–3000	0.9966	46	150
	Wheat	y = 230.94x − 866.12		0.9991		
	Maize	y = 103.05x − 167.52		1.0000		
	Barley	y = 237.94x − 866.12		0.9999		
FB2	Solvent	y = 761.01x − 16822	150–3000	0.9945	46	150
	Wheat	y = 493.7x − 4122.5		0.9966		
	Maize	y = 209.36x − 598.27		0.9999		
	Barley	y = 578.72x − 7184.9		0.9985		
ZEA	Solvent	y = 91.077x + 3.8326	30–600	0.9997	9	30
	Wheat	y = 70.491x + 72.751		0.9992		
	Maize	y = 26.71x + 35.167		0.9993		
	Barley	y = 70.654x − 22.304		0.9999		
T-2	Solvent	y = 696.5x − 134.59	10–200	0.9996	3	10
	Wheat	y = 757.94x + 36.431		0.9996		
	Maize	y = 267.43x − 69.004		1.0000		
	Barley	y = 760.5x + 36.722		0.9999		
HT-2	Solvent	y = 98.501x − 56.058	10–200	0.9975	3	10
	Wheat	y = 97.296x + 87.819		0.9997		
	Maize	y = 39.128x + 12.603		0.9996		
	Barley	y = 115.99x + 58.863		0.9999		
OTA	Solvent	y = 1049.8x − 52.161	1.0–20	0.9979	0.30	1.0
	Wheat	y = 853.76x − 68.076		0.9992		
	Maize	y = 375.68 − 42.071		0.9991		
	Barley	y = 889.28x − 14.52		0.9964		

**Table 7:** Method recovery (R%) and repeatability of measurement (RSD_M_%) and sample preparation (RSD_P_%) in maize using solvent and matrix-matched calibration.

	Maize																			
	Spike Level 1						Spike Level 2						Spike Level 3							
	R%		RSD_M_%		RSD_P_%		R%		RSD_M_%		RSD_P_%		R%		RSD_M_%		RSD_P_%		Average R%	
Analyte	Solvent	Matrix	Solvent	Matrix	Solvent	Matrix	Solvent	Matrix	Solvent	Matrix	Solvent	Matrix	Solvent	Matrix	Solvent	Matrix	Solvent	Matrix	Solvent	Matrix
AFB1	101.0	109.9	5.4	5.9	6.9	10.6	73.3	101.7	3.5	2.0	5.8	3.2	75.9	96.0	1.8	1.7	5.2	4.1	83.4	102.5
AFB2	88.8	85.3	8.4	12.6	9.4	15.9	75.6	106.8	6.3	6.9	9.4	15.9	84.5	98.2	6.4	4.7	12.6	13.9	83.0	96.8
AFG1	98.5	81.8	8.0	10.4	13.7	13.6	82.1	83.1	2.4	3.2	3.9	4.1	94.5	96.2	3.7	4.1	5.0	5.2	91.7	87.0
AFG2	95.4	115.7	7.1	8.9	8.9	9.7	83.7	102.1	4.6	6.8	5.7	8.4	96.0	115.7	4.9	4.7	8.4	5.4	91.7	111.2
DON	69.0	66.4	3.5	6.4	5.6	7.0	119.6	95.4	2.6	2.2	3.6	4.1	91.3	99.7	2.6	2.9	4.9	4.1	93.3	87.2
FB1	169.0	95.2	1.7	1.6	3.3	3.1	349.7	104.4	1.1	1.2	2.3	2.4	800.6	108.8	3.9	2.3	1.6	2.8	439.8	102.8
FB2	180.3	104.7	1.4	1.5	3.5	1.6	151.9	102.8	1.8	1.9	3.1	3.0	164.6	107.8	1.0	1.7	2.8	3.2	165.6	105.1
ZEA	79.1	81.5	2.6	3.1	4.0	3.5	70.4	78.0	1.2	1.4	2.7	2.5	91.5	100.8	1.2	1.2	2.8	2.8	80.3	86.8
T-2	93.0	99.7	3.9	2.5	7.3	4.8	117.9	98.2	1.9	4.1	3.1	4.1	114.3	107.0	2.0	2.0	2.7	2.8	108.4	101.6
HT-2	79.4	99.4	9.2	6.4	14.9	8.6	102.0	81.5	4.1	4.4	6.3	6.4	122.6	99.1	4.4	3.3	7.4	4.1	101.3	93.3
OTA	108.7	112.8	11.4	5.4	19.4	6.7	36.6	102.1	20.0	8.4	14.2	7.2	60.7	93.9	9.3	9.7	9.1	6.1	68.7	102.9

**Table 8:** Method recovery (R%) and repeatability of measurement (RSD_M_%) and sample preparation (RSD_P_%) in wheat using solvent and matrix-matched calibration.

	Wheat																			
	Spike Level 1						Spike Level 2						Spike Level 3							
	R%		RSD_M_%		RSD_P_%		R%		RSD_M_%		RSD_P_%		R%		RSD_M_%		RSD_P_%		Average R%	
Analyte	Solvent	Matrix	Solvent	Matrix	Solvent	Matrix	Solvent	Matrix	Solvent	Matrix	Solvent	Matrix	Solvent	Matrix	Solvent	Matrix	Solvent	Matrix	Solvent	Matrix
AFB1	78.3	99.7	2.7	4.6	6.6	5.4	92.4	102.9	4.7	2.3	5.7	4.0	81.2	94.6	1.6	2.2	3.6	4.5	84.0	99.1
AFB2	99.6	100.6	6.6	5.8	9.4	6.2	86.0	115.7	6.6	5.8	9.4	6.2	88.4	87.3	10.3	8.7	12.6	13.9	91.3	101.2
AFG1	95.7	106.6	10.2	5.4	8.5	7.8	84.7	104.0	3.1	3.8	8.5	6.3	79.5	104.0	4.8	4.5	6.7	6.9	86.6	104.9
AFG2	91.4	100.1	3.5	4.6	5.2	6.9	79.9	93.9	5.4	5.8	5.4	5.8	81.8	96.8	5.1	5.3	6.5	5.7	84.4	96.9
DON	66.6	108.7	4.1	2.2	6.1	4.6	93.4	114.2	2.9	2.0	4.8	4.0	79.7	110.5	1.7	1.1	3.3	4.2	79.9	111.1
FB1	226.4	101.9	1.7	1.3	2.3	1.8	237.5	106.1	1.6	1.1	3.4	3.6	200.4	96.2	1.7	1.0	5.3	3.5	221.4	101.4
FB2	207.6	112.1	1.1	2.2	2.3	3.4	166.1	97.5	1.5	1.6	3.7	3.3	144.5	91.5	1.3	1.0	3.6	2.5	172.7	100.4
ZEA	73.9	97.3	1.6	2.6	5.1	4.0	85.2	100.3	1.9	1.1	2.5	1.4	89.2	94.0	1.1	1.1	30.0	2.9	82.8	97.2
T-2	115.6	92.0	3.7	5.0	6.4	2.8	99.6	99.6	1.8	3.4	4.1	7.4	99.9	100.2	2.7	2.5	2.5	3.1	105.0	97.3
HT-2	110.3	98.8	6.2	5.0	11.1	5.8	100.0	89.4	7.4	9.3	9.8	17.4	74.0	92.4	8.0	9.2	12.9	13.8	94.8	93.5
OTA	97.2	102.5	6.4	7.7	17.3	7.2	97.1	93.6	3.7	8.0	3.2	6.0	94.8	86.9	4.3	8.4	1.9	6.0	96.4	94.3

**Table 9:** Method recovery (R%) and repeatability of measurement (RSD_M_%) and sample preparation (RSD_P_%) in barley calculated using solvent and matrix-matched calibration.

	Barley																			
	Spike Level 1						Spike Level 2						Spike Level 3							
	R%		RSD_M_%		RSD_P_%		R%		RSD_M_%				R%		RSD_M_%		RSD_P_%		Average R%	
Analyte	Solvent	Matrix	Solvent	Matrix	Solvent	Matrix	Solvent	Matrix	Solvent	Matrix	Solvent	Matrix	Solvent	Matrix	Solvent	Matrix	Solvent	Matrix	Solvent	Matrix
AFB1	84.7	109.6	7.0	4.9	7.5	6.2	85.8	99.0	4.7	4.4	5.0	4.5	85.6	91.7	2.9	2.7	5.0	4.6	85.4	100.1
AFB2	99.6	97.8	6.3	7.9	7.0	12.8	85.0	99.2	7.4	7.2	8.2	6.7	98.6	103.9	6.3	6.3	7.5	7.7	94.4	100.3
AFG1	85.0	103.4	3.6	8.9	6.5	8.5	87.2	98.5	4.2	2.4	5.3	3.6	79.7	89.3	2.5	2.1	4.8	4.0	84.0	97.1
AFG2	116.4	112.9	5.1	4.4	6.4	6.9	81.5	112.8	5.2	5.8	8.9	6.2	101.2	105.1	5.2	5.0	6.2	5.5	99.7	110.3
DON	86.3	97.2	3.2	5.2	4.4	5.7	73.2	83.2	1.9	2.5	4.9	7.5	72.0	82.5	2.5	2.3	3.4	3.9	77.2	87.6
FB1	169.0	95.2	2.5	3.0	6.4	6.4	157.6	89.9	2.6	1.2	3.6	4.6	157.1	83.1	4.6	1.7	3.0	2.7	161.2	89.4
FB2	180.3	104.7	1.6	3.1	1.9	3.5	139.7	82.6	1.8	0.9	2.3	3.3	140.9	80.7	2.2	1.2	2.5	2.8	153.6	89.3
ZEA	88.3	105.0	1.3	2.0	5.8	9.1	99.1	92.2	1.6	2.0	3.0	6.4	87.1	78.7	1.4	1.3	3.0	2.7	91.5	92.0
T-2	97.6	100.1	6.0	4.9	6.7	4.9	107.3	89.3	1.9	2.1	3.5	2.1	104.4	78.3	1.5	1.5	3.3	3.1	103.1	89.2
HT-2	100.7	92.9	3.8	11.0	5.8	17.6	95.9	78.8	6.2	3.8	7.3	7.1	102.4	101.9	1.7	2.2	2.9	2.2	99.7	91.2
OTA	97.2	102.5	6.4	7.7	17.3	7.2	61.8	52.1	8.6	5.1	6.3	6.5	40.6	35.1	9.8	12.0	5.0	6.1	66.5	63.2

**Table 10:** PT results for mycotoxins in food and feed matrices.

PT Scheme	Matrix	Analyte	z-Score
Romer Labs CSSMY0150-M18411AF	Maize	AFB1	−1.20
		AFB2	−0.50
		AFT	−1.10
Romer Labs CSSMY014-M18161DZ	Wheat	DON	0.30
		ZEA	1.60
Bipea 03-0531	Barley	AFB1	−1.48
		AFB2	0.00
		AFG1	−0.08
		AFG2	0.35
		T2	−0.21
		HT2	0.15
Fapas 04351	Cereal-based feed (wheat)	AFB1	0.00
		OTA	0.60
		ZEA	0.30
Bipea 12-3931	Cereal-based baby food (maize)	OTA	0.42
		DON	0.58
		T2	0.84
		HT2	0.12
		T2 + HT2	0.70
		ZEA	−0.11
		FB1	2.41
		FB2	0.98
		FB2 + FB2	1.70
Bipea 13-3931	Cereal-based baby food (maize)	OTA	1.48
		DON	0.67
		T2	1.06
		HT2	0.35
		T2 + HT2	1.18
		ZEA	0.57
		FB1	−0.39
		FB2	−0.85
		FB2 + FB2	−0.83
Bipea 02-4731	Buckwheat flour	AFB1	−0.17
		AFB2	0.38
		AFG1	−1.00
		T-2	0.70
		HT-2	0.11
Bipea 01-5131	Rye	AFB1	0.00
		AFB2	−0.25
		AFG1	−0.20
		AFG2	−0.45
		T-2	−0.52
		HT-2	−0.30
